# Prevalence and Associated Factors of Posttraumatic Stress Symptoms and Stigma among Health Care Workers in Contact with COVID-19 Patients

**DOI:** 10.18502/ijps.v15i4.4303

**Published:** 2020-10

**Authors:** Atefeh Zandifar, Rahim Badrfam, Nami Mohammadian Khonsari, Mohammad Reza Mohammadi, Hamid Asayesh, Mostafa Qorbani

**Affiliations:** 1 Cardiovascular Research Center, Shahid Rajaei Educational and Medical Center, Alborz University of Medical Sciences, Karaj, Iran.; 2 Social Determinants of Health Research Center, Alborz University of Medical Sciences, Karaj, Iran.; 3 Department of Psychiatry, Roozbeh Hospital, School of Medicine, Tehran University of Medical Sciences, Tehran, Iran.; 4 Alborz University of Medical Sciences, Karaj, Iran.; 5 Psychiatry and Psychology Research Center, Roozbeh Hospital, Tehran University of Medical Sciences, Tehran, Iran.; 6 Department of Medical Emergencies, Qom University of Medical Sciences, Qom, Iran.; 7 Non-Communicable Diseases Research Center, Alborz University of Medical Sciences, Karaj, Iran.; 8 Chronic Diseases Research Center, Endocrinology and Metabolism Population Sciences Institute, Tehran University of Medical Sciences, Tehran, Iran.

**Keywords:** *COVID-19*, *Comorbidity*, *Health Care Workers*, *Prevalence*, *Posttraumatic Stress Symptoms*, *Stigma*

## Abstract

**Objective:** The global spread of COVID-19 has caused great psychological stress in health care workers (HCWs). This study aims to assess the prevalence and associated factors of posttraumatic stress symptoms (PTSS) and stigma among health care workers (HCWs) who are involved in treating COVID-19 patients. This study was conducted as part of studies related to assessing the mental health status of HCWs in Iran during the COVID-19 pandemic.

**Method:** Overall, in this cross sectional study, 894 HCWs working in 9 general hospitals in Alborz province, Iran, in the field of diagnostic and treatment care of patients with COVID-19 were selected using multistage sampling method. PTSS and its subscales, including intrusion, avoidance, and hyper vigilance were obtained based on the Posttraumatic Stress Disorder-8 Item validated questionnaire. Information on Stigma's perception among HCWs was also obtained based on a questionnaire adopted from the HIV Stigma Scale. Predictors of PTSS and Stigma's perception among HCWs were assessed using multivariate logistic regression analysis.

**Results: **Overall, 71.4% of the participants were women and 46.9% were front line staff. The prevalence of intrusion, avoidance, and hyper vigilance symptoms was 44.2% (95% CI: 40.8-47.6), 31. 8% (95% CI: 28.8-35.0), and 37.7% (95% CI: 34.5-41.0), respectively. A strong and positive significant correlation was found between stigma score with PTSD total score (coefficient: 0.83) and its components. In multivariate logistic regression model, female gender was associated with intrusion (OR: 1.46, 95% CI: 1.03-2.06) and avoidance (OR: 1.66, 95% CI: 1.147-2.417) and working in frontline increased the odds of intrusion (OR: 1.45, 95% CI: 1.06-1.97) and hyper vigilance (OR: 1.41, 95% CI: 1.03-1.91).

**Conclusion: **The prevalence of PTSS is high among HCWs during COVID-19 pandemic and it is associated with some demographic characteristics of HCWs. This situation should be considered by health policymakers so that while trying to control the disease, the mental health status of this group of personnel should be given much attention.

The global spread of COVID-19 has caused much psychological stress in health care workers (HCWs); thus, in the literature, attention has been paid to their mental health (1). Several studies have evaluated the immediate impressions of COVID-19 on HCWs and have described acute stress, depression, and anxiety as their mental health difficulties. In most studies, the severity of these symptoms and disorders among HCWs during the COVID-19 pandemic has been reported to be relatively high (2, 3). 

Many HCWs caring for patients with COVID-19 report extensive strain conditions due to stressful situations accompanied with depression and anxiety. The severity of these symptoms is influenced by various factors such as age, gender, occupational status, and the level of contact with patients with COVID-19 (4). A group of these HCWs who are in front line with patients with COVID-19 have a greater sense of fear and stress and are more likely to have psychological disorders (2). In addition to the severe stress that HCWs face in hospital settings, they are also prone to posttraumatic stress symptoms (PTSS). Death of patients, the lack of relevant facilities and ethical issues, and the persistence of these problems, along with the lack of action to receive mental health services by HCWs for a variety of reasons, including stigma, denial, conflicting time demands, and the like can lead to chronic disorders (5). In fact, stigma, in the form of a negative attitude toward people with certain conditions or illnesses (6), may prevent HCWs from pursue diagnostic and therapeutic measures related to psychiatric symptoms. This can lead to serious psychiatric disorders such as depression and posttraumatic stress disorder (PTSD) (7). 

Because the disease has a high mortality rate, some experts consider this disease to be a life-threatening disease and a traumatic event and consider HCWs and stigmatized groups to be prone to PTSD (8). The death of patients and the fear and anxiety of the situation and the uncertainty of the condition can lead to acute stress disorder, which has the potential to become PTSD. On the other hand, poor knowledge of the virus and the conditions under which it can be spread can be very anxious and can be a prelude to acute stress disorder (ASD) or PTSD (9). In fact, the unpredictability of the condition as well as the fear of the disease and its dangers is one of the most stressful situations (1). On the other hand, the sever acute respiratory syndrome (SARS) epidemic experience has shown that fear, uncertainty, and stigma can lead to psychological distress, especially among HCWs in frontline situations (10). 

Huang et al reported high levels of stress disorder among HCWs and reported that about a quarter of HCWs had symptoms of PTSD. They reported the disorder among female staff more than men (11). Wu et al, in a comparative study of a group of HCWs who were in direct contact with patients with COVID-19 with the control group of hospital staff, stated that the rate of mental health disorders in this group was higher than the control group. They said staff fighting covid-19 were exposed to psychological symptoms and sleep problems that interacted with each other (12). 

Stigma towards staff in some situations can occur as others may think they can transmit coronavirus, depending on their job status and leading them to be excluded from society. Feelings of depression and uselessness are sometimes followed by quitting a job, which in itself can be an important factor in creating psychological distress (13). Despite some individual differences, people undergoing stigma experience pervasive stress that can lead to feelings of social worthlessness. However, the boundaries of stigma and the resulting psychological effects seem to go beyond social constraints and moral transgressions (14). 

In this study, we examined the status of PTSS and its components, including intrusion, avoidance, and hypervigilance in the context of staff exposure to patients with COVID-19. In addition, given the semantic breadth of the stigma, in this study, we examined its status among HCWs who were exposed with patients with COVID-19 and evaluated the relationship between these two important components. This study was conducted as part of studies related to assessing the mental health condition of health care workers in Iran during the COVID-19 pandemic (15). 

The aim of this study was to identify HCWs at risk or with symptoms of PTSS and stigma related to COVID-19 and to perform treatment and related follow-up. Also, the aim of this study was to obtain epidemiological information on these symptoms for related planning in other similar centers.

## Materials and Methods

***Study Design***

A total of 894 HCWs engaged in the field of diagnostic and treatment of COVID-19 patients in 9 general hospitals in Alborz province, Iran, were selected for this cross-sectional study using available sampling method. The study was conducted from March 20 to April 3, 2020. Participants worked in various internal medicine and infectious wards and in the intensive care units (ICU) of the hospitals. They were physicians, nurses, and technicians, and their employment status was in the form of formal employment, informal employment, and medical resident.

Some of them were in the front line (Corona emergency or infectious wards or ICU) and others were in indirect contact with patients. They were in the hospital full-time or part-time. The questions in the form of a questionnaire were provided electronically to the participants. Participating in the study was completely voluntary. Participants were informed that they could pull out the study at any time if they wanted. It was also stated that by providing their contact number, they can receive diagnostic or therapeutic services based on the results of the study. All services were provided free of charge by a predetermined psychiatrist or psychologist.

***Inclusion and Exclusion Criteria***

The participants in the study were HCWs who worked in the internal medicine and infectious wards and intensive care units of the general hospitals of Alborz province in Iran. (All participants aged over 18 years.). They were in contact with patients with COVID-19 and worked full-time or part-time. They were present at the hospital on various daily or night shifts or rotations, and if they wished, they would enter the research project.

***Data Collection***

Participants' demographic information, including age, gender, employment status, occupation, level of education, and type of contact with patients with COVID-19, was obtained based on the information requested from patients before completing the study questionnaires. Information on PTSS was also obtained based on the Posttraumatic Stress Disorder 8-Item questionnaire. Information on Stigma's perception among HCWs was obtained based on a questionnaire obtained from a questionnaire adopted from the HIV Stigma Scale. 

***Procedure***

***The Site***

The study was conducted at 9 general hospitals in Iran's Alborz province, including all hospitals covered by Alborz University of Medical Sciences. This province is one of the most populous provinces in Iran (16). HCWs working in the isolated internal medicine and infectious wards and the intensive care unit for patients with COVID-19 were evaluated.

***Selection of Participants***

Questionnaires were sent to 971 HCWs, and the response rate was 92% (The total number of final questionnaires evaluated was 894). They entered the study based on the inclusion criteria and through the proportional random sampling method. Based on how the questionnaires were filled in, a number of missing data were obtained in different items, which were related to the incomplete answers of the participants.

***Scale***

***Posttraumatic Stress Disorder 8-Item Questionnaire***

Possible psychological effects following various traumas need to be assessed and screened to identify risk factors and related prevention and treatment measures at the appropriate time. The use of simple short screening instruments in the field of trauma, which has good psychometric properties based on the balance of sensitivity and specificity, is important due to the special conditions of presence in trauma-related environments. Posttraumatic stress disorder 8-item questionnaire has been validated by Hansen et al in 3 samples related to different traumas, and in the fourth example, its test-retest reliability has been evaluated and confirmed. The Cronbach's alpha in the internal consistencies of 3 samples equaled to 0.83, 0.84, and 0.85, which is appropriate (17). With the help of this questionnaire, it is possible to make a proper assessment of the status of 3 domains related to PTSD (intrusion, avoidance, and hyperarousal). The PTSD-8 scale has 8 items that comprise 7 PTSD marks associated with ICD-11. Each item has a 4-point Likert scale (1 = not at all, 4 = very often). Having at least 1 item within each PTSD symptom cluster (intrusion, avoidance, hyperarousal) with a score of 3 or higher can be considered as a possible PTSD. In a study by Andersen et al, on the validation of the PTSD-8 Scale in chronic pain patients, Cronbach's alpha for the total scale was 0.84, which was satisfactory (18). This questionnaire has also been validated in Iran. To this end, Asayesh et al. examined the prevalence of PTSD among nurses exposed to patients with COVID-19. In this study, this questionnaire was first translated from English to Persian and then the Persian version was back translated into English by another person, and finally after reviewing the 2 versions, the final Persian version was prepared. Cronbach's alpha was 0.86 and the correlation coefficient in the test-retest method was 0.83 (Unpublished manuscript). 

***The Perception of Stigma (Questionnaire Adopted from the HIV Stigma Scale)***

This questionnaire focuses on experiences and feelings as to how HCWs feel the stigma in the context of COVID-19 outbreak. In some studies, to assess the status of the perception of stigma among study participants, based on theoretical domains, including stereotypes, discrimination, shame, and social isolation, their status is assessed (19). Sometimes a questionnaire related to the perception of stigma related to another disease (such as the HIV Stigma Scale in our study) is used for this purpose (20). The questionnaire used in this study was a 22-item modified Stigma-related questionnaire for HIV patients (21). Each item has a 5-point Likert scale. 

This questionnaire is based on two studies. The internal consistency of the first 2 subscales was good with the Cronbach’s alpha of 0.82 and 0.81, respectively. The internal consistency of the third subscale was acceptable, as its Cronbach's alpha was equal to 0.68.

This questionnaire has been translated into Persian and the validity and reliability of the Persian version has been further evaluated. The Cronbach's alpha coefficient was 0.75 for all items (α = 0.75). The answer to each item is one of the options "strongly disagree", "disagree", "no idea", "agree" and "strongly agree" (22). 

***Statistical Methods***

All analyses were done using SPSS software version 16. The normal distribution of continuous variables was assessed using the Kolmogrov-Smirnov test. Variables with and without normal distribution were identified as mean (standard deviation (SD)) and median (inter quartile range (IQR)), respectively. Qualitative variables are presented as frequency and percentages. The Mann-Whitney U and Kruskal-Wallis tests were used to compare the PTSD and its subscales (intrusion, avoidance and hyper vigilance) and stigma scores across demographic characteristics variables. Spearman correlation coefficient was used to compare the correlation between PTSD and its subscales with stigma scores. Multivariate logistic regression analysis was used to determine the associated factors of intrusion, avoidance and hypervigilance among HCWs. To control the interfering variables and eliminate the confounding factors, logistic regression analysis was used. The result of logistic regression analysis was presented as odds ratio (OR) and 95% confidence interval (CI). If p <0.05, statistical observation was considered significant.

***Ethical Considerations***

The ethics committee of Alborz University of Medical Sciences approved this research project on 06/04/2020 (IR.ABZUMS.REC.1399.01). Participants were informed about the components of the study and its objectives upon entering the study. Participants were asked to provide their contact numbers, if desired, and to be further investigated as necessary and based on the information obtained from the questionnaire on psychological or psychiatric issues. They were also informed that the services provided to them in this area are completely voluntary and free. All components of the Helsinki Declaration were considered in this study.

Participants in the project were assured that all information provided by them would be confidential and would not be shared with any individual or group.

## Results

Overall, 71.4% of the participants were women and 46.9% in front line staff. In terms of hiring status, 44.9% were in officially employed and 49.6% had non-official status, and 4.6% were medical residents. Bachelor education had the highest rate of education at 65.8% and 18.8% had a higher education level than bachelor’s degree. Also, 60.7% of the participants were nurses, 8.9% physicians, and rest technicians. The prevalence of intrusion, avoidance and hypervigilance symptoms was 44.2% (95% CI: 40.8-47.6), 31.8% (95% CI: 28.8-35.0), and 37.7% (95% CI: 34.5-41.0), respectively (Table 1). 

Intrusion was significantly more prevalent in females (p value = 0.01), people with formal employment (p value <0.001), and in front line staff (p value = 0.04). This situation was achieved in the field of avoidance in the form of more prevalence among females (p value < 0.001), staff with higher education than bachelor's degree (p value = 0.01), and paramedics (p value <0.001). Hypervigilance was also more common in medical residents (p value < 0.01) in front line staff (p value < 0.004), and physicians (p value < 0.001) (Table 2). 

Based on median and interquartile range (IQR), intrusion was significantly higher in females (p-value < 0.001), in staff with higher bachelor's degree (p value = 001), in non-official employment conditions (p value <0.001), in personnel in situ front line (p value = 0.03), in people with a technician job status (p-value <0.001), and in those younger than 30 years. Also, avoidance was significantly higher in female staff (p value <0.001), in those with higher education than bachelor's degree (p value < 0.002), and in those with non-formal employment (p value < 0.001). Hyper vigilance was more common in female staff (p value = 0.001), medical residents (p value <0.001), in frontline personnel (p value = 0.004), and technicians (p value = 0.002). In overall scaling, the total posttraumatic stress score was higher in females (p value <0.001), in staff with higher educational level than bachelor's degree (p-value = 0.004), in those with non-official employment status (p-value <0.001), those with technician job status (p-value = 0.01), and those older than 30 years (p-value = 0.05). Stigma was more prevalent among females (p value = 0.01), medical assistants (p value = 0.03), frontline staff (0.006), and physicians (p value < 0.04) (Table 3). 

Correlation between stigma score and PTSD total score and its subscales is presented in Table 4. There was a strong and positive significant correlation between stigma score with PTSD total score (coefficient: 0.83) and its components, including intrusion (coefficient: 0.72), avoidance (coefficient: 0.69), and hypervigilance (coefficient: 0.76) among HCWs.

In multivariate logistic regression model, female gender was associated with intrusion (OR: 1.46, 95% CI: 1.03-2.06) and avoidance (OR: 1.66, 95% CI: 1.147-2.417) and working in frontline increased the odds of intrusion (OR: 1.45, 95% CI: 1.06-1.97) and hypervigilance (OR: 1.41, 95% CI: 1.03-1.91). Moreover, non-official HCWs had lower odds of intrusion and avoidance compared to official workers (Table 5). 

**Table 1:** Frequency and Missing Percentage of Demographic and Psychiatric Characteristics (n=894)

**Variable**		**Number**	**percent**	**Missing %**
Sex	Male	254	28.4	0.2
	Female	638	71.4	
Frontline staff	No	475	53.1	0
	Yes	419	46.9	
Hiring status	Official	401	44.9	1
	Non official	443	49.6	
	Resident	41	4.6	
Education	=<Diploma	124	13.9	1.6
	Bachelor	588	65.8	
	> Bachelor	168	18.8	
Occupation	physician	80	8.9	2
	nurse staff	543	60.7	
	technician	253	28.3	
Age group (yea)	<30y	296	33.1	1.6
	31-40	374	41.8	
	>40	210	23.5	
Intrusion	No	476	53.2	4.6
	Yes	377	42.2	
Avoidance	No	601	67.2	1.3
	Yes	281	31.4	
Hyper vigilance	No	549	61.4	1.3
	Yes	333	37.2	

**Table 2:** Prevalence of Intrusion, Avoidance and Hypervigilance According to Demographic Characteristics

**severity ** **category**	**Sex**		**education**			**Hiring**			**job duration**			**front line**		**occupation**			**age**		
	Male	female	=<diploma	=<license	>license	official	non official	student	5>	6 to 10	10<	Yes	no	physician	nurse	Technic ian	30>	30- 40	40<
Intrusion	92 (37.4)	284 (46.9)	55 (44.7)	250 (44.5)	65 (41.1)	198 (52.7)	155 (36.3)	16 (39)	210 (43.8)	49 (47.6)	15 (36.6)	193 (47.8)	184 (41)	30 (38.5)	235 (45)	100 (42.2)	119 (40.9)	169 (47.6)	87 (43.9)
p-value	0.01*		0.74			0.001>*			0.48			0.04*		0.48			0.23		
avoidance	59 (23.5)	222 (35.3)	26 (21)	194 (33.5)	57 (34.3)	148 (37.7)	113 (25.7)	18 (43.9)	168 (33.9)	33 (32)	9 (22)	132 (31.9)	149 (31.8)	31 (38.8)	173 (32.3)	71 (28.5)	91 (30.7)	122 (33.2)	67 (32.5)
p-value	0.001>*		0.01*			0.001>*			0.28			0.98		0.21			0.79		
hyper vigilance	82 (33.1)	251 (39.7)	40 (32.8)	218 (37.5)	70 (42.2)	157 (39.7)	148 (33.9)	22 (53.7)	206 (41.4)	41(39.8)	14 (34.1)	177 (42.8)	156 (33.3)	43 (53.8)	209 (38.8_	74 (29.8)	113 (38.6)	142 (38.3)	71 (34.5)
p-value	0.06		0.26			0.01*			0.65			0.004*		0.001>*			0.59		

**Table 3:** Median (IQR) of the Total and Subscale PTSS Score According to Demographic Characteristics

**severity ** **category**		**intrusion**		**p value**	**Avoidance**		**p value**	**hyper vigilance**		**p value**	**PTSS total**		**p value**	**Stigma**		**P value**
		**Median**			**Median**	**IQR**		**Median**	**IQR**		**Median**	**IQR**		**Median**	**IQR**	
sex	Male	8	4	0.001>*	4	3	0.001>*	4	2	0.001*	16	9	0.001>*	26	15.25	0.01*
	Female	8	5		4	3		4	2		19	9		27	17	
education	=<diploma	8	6	0.01*	4	3	0.002*	4	2.5	0.19	16	12	0.04*	23	13.5	0.74
	=<license	8	4		4	3		4	3		19	7.5		26	17	
	>license	10	6.75		5	3.25		5	2.75		19	12.25		29	21	
hiring	Official	8	3.25	0.001>*	5	2	0.001>*	5	2	0.001>*	18.5	6.25	0.001>*	29	17.25	0.003*
	non-official	8	5		4	4		4	3		19	11		22	15	
	resident	7	3		4	2		5	3		16	5.5		29	20	
job duration	5>	8	5	0.28	5	3	0.23	5	2	0.84	19	9	0.4	26	17	0.60
	6 to 10	8	6		4	3.25		4	3.25		17	11		28	17	
	>10	8	5.5		5	2		4	3		18	11		21	19	
front line	Yes	8	5	0.03*	5	3	0.54	5	4	0.04*	19	10	0.08	28	17	0.006*
	No	8	5		4	2		4	1		16	7		25	17	
occupation	Physician	6	5	0.001>*	3	2.5	0.12	4	1.5	0.002*	15	7	0.01*	29	19.5	0.04*
	Nurse	8	4		4	3		4	3		19	8.5		26	17	
	Technician	8.5	5.75		5.5	2		5	3		19	11		22	32	
age	30>	8	5	0.01*	4	3.75	0.24	4	3	0.19	16	11.25	0.05*	25	16	0.58
	30-40	8	4.25		4	2.25		4	2.5		19	9.25		28	16	
	40<	8	4.75		5	2		4	1		18.5	7		25	17	

**Table 4:** Correlation between PTSD Total and Subscale Score with Stigma Score

	**PTSD total score**	**Intrusion score**	**Avoidance score**	**Hypervigilance score**	**Stigma score**
PTSD total score	1.000	0.918^*^	0.849^*^	0.822^*^	0.836^*^
Intrusion score	0.918^*^	1.000	0.662^*^	0.630^*^	0.724^*^
Avoidance score	0.849^*^	0.662^*^	1.000	0.614^*^	0.693^*^
Hyper vigilance score	0.822^*^	0.630^*^	0.614^*^	1.000	0.767^*^
Stigma score	0.836^*^	0.724^*^	0.693^*^	0.767^*^	1.000
*Statistically significant					

**Table 5:** Association of Intrusion, Avoidance and Hypervigilance with Demographic Characteristics in Logistic Regression Analysis

	**variable**	**Intrusion**		**Avoidance**		**Hypervigilance**	
		case with outcome/ total cases	adjusted OR	case with outcome/ total cases	adjusted OR	case with outcome/ total cases	adjusted OR
sex	male	92/246	1	59/251	1	82/248	1
	female	284/605	1.465 (1.037-2.068)*	222/629	1.665 (1.147-2.417)*	251/632	1.341 (0.944-1.905)
education	=<diploma	55/123	1	26/124	1	40/122	1
	-<license	250/562	0.821 (0.523-1.288)	194/579	1.346 (0.805-2.248)	218/581	1.052 (.659-1.682)
	>license	65/158	0.639(0.341-1.195)	57/166	1.032 (0.524-2.032)	70/166	0.788 (0.412-1.510)
hiring	official	198/376	1	148/393	1	157/395	1
	non official	155/427	0.5 (.364-.685)*	113/439	0.653 (0.471-0.907)*	148/437	0.841 (0.613-1.154)
	student	16/41	0.819 (0.296-2.266)	18/41	1.222 (0.439-3.402)	22/41	0.777 (0.288-2.098)
age	30>	119/291	1	91/296	1	113/293	1
	30-40	169/355	1.242 (0.875-1.763)	122/368	1.051 (0.728-1.518)	142/371	1.126 (0.793-1.598)
	40<	87/198	1.087 (0.715-1.653)	67/206	1.008 (0.651-1.561)	71/206	0.861 (0.562-1.319)
occupation	physician	30/78	1	31/80	1	43/80	1
	nurse	235/522	0.932 (0.396-2.192)	173/536	0.615 (0.257-1.472)	209/539	0.33 (0.141-0.770)*
	technician	100/237	1.143 (0.489-2.675)	71/249	0.639 (0.268-1.523)	74/248	0.273 (0.117-0.638)*
front line	no	184/449	1	149/468	1	156/468	1
	yes	193/404	1.452 (1.068-1.974)*	132/414	1.015 (0.737-1.397)	177/414	1.410 (1.038-1.915)

## Discussion

In our study more than two-thirds of the participants were female HCWs, which was commensurate with the gender distribution of the staff. Due to the different proportions of the physician staff to nurses and technicians, the number of physicians participating in the study was lower than other occupational groups.

In the SARS epidemic experience, as a traumatic experience in some respects, similar to COVID-19, one of the most common psychiatric disorders was PTSD, which continued even months later. Its cumulative prevalence in the general population was estimated at nearly 50%, and one of the most vulnerable groups in the field was HCWs. Also, stigmatization was one of the consequences of this outbreak and the accompanying psychiatric disorders (23). In this epidemic, the overall prevalence of PTSD among HCWs was estimated at about 10%, and factors such as quarantine, front line status, and close friends and relatives with SARS increased the risk by 2 to 3 times. According to some researchers, the perception of risks associated with SARS was associated with severity of symptoms in HCWs. They described altruistic acceptance of work-related risks in these situations as having a negative relationship with the severity of symptoms for HCWs (24). 

In our study, more than a third of HCWs had PTSD symptoms, indicating a high rate of these symptoms among HCWs. Iran was one of the countries that, after China, experienced the most sudden spread of the disease with the highest mortality rate per affected population, especially in the early stages of the disease (25, 26). The inappropriate economic situation, especially in recent years, has been a factor in reducing the ability to meet the needs of equipment for personal care and complex medical equipment (27). Thus, in Iran, at the time of onset of spread of the disease, COVID-19 was associated with increased fear and anxiety, which could lead to symptoms such as PTSD and its dimensions (28, 29)These points can also have a significant impact on the development of stigma among HCWs in our study (30). 

 As with SARS studies, in our study, the prevalence of PTSD was higher among HCWs, which were in front line position. In addition, in our study, stigma was more prevalent among this group of HCWs. This can reflect the greater effectiveness of this group due to the higher exposure and more stressful working conditions, which is associated with increased anxiety. It seems traumatic experiences can become chronic and destructive. 

Anxiety, anger, and grief seem to be the common experiences of all those facing loss of life. These can lead to mental health damage and long-term consequences (31). Such experiences are exacerbated by the spread of infectious diseases associated with increased mortality or short-term or long-term disability (5). This is also the case with the potential long-term consequences of viral disease pandemics and can be associated with worrying consequences for mental health (32) and medical condition (33). 

Huang et al, in their assessment of frontline HCWs, reported the prevalence of stress disorder of 27.39%, with a higher prevalence in females. They noted the high prevalence of these disorders among frontline HCWs and emphasized the necessity of consideration mental health and psychological skills training (11). It is important to pay attention to the mental health status of the HCWs, especially in the context of stress and anxiety problems. This is even more important in the case of epidemics. In such cases, the use of a variety of intervention methods, including social media assistance and the use of tele-psychiatry, is helpful (34, 35). Also, according to various studies, the use of social capital in this field can play an effective role in reducing mental health disorders (36). 

Shortly after the outbreak of COVID-19, Liu et al. stated the prevalence of posttraumatic stress symptoms (PTSS) among residents of Wuhan County at the height of the disease to be7%. In re-experiencing and hyperarousal domains, the prevalence was clearly higher in females. They considered it necessary to provide professional and effective mental health services to achieve psychological wellbeing (37). 

Yin et al continued to evaluate the prevalence of posttraumatic stress symptoms (PTSS) among HCWs who took part in the study from different provinces of China and stated that its prevalence was 3.8%, which was more common among women. This study was performed sometime after the previous study (Liu et al) and there was a relative improvement in epidemic control (38). As can be seen in our study, the prevalence of PTSS is higher among females than males. This may indicate that women may be more vulnerable to environmental stressors, especially in the context of epidemics. On the other hand, part of the cause of the higher prevalence of the symptoms in females can be due to changes in the levels of ovarian hormones in them during exposure to these stressful environmental conditions. Another part may be related to the probable further negative cognitive changes among females in this situation (37). 

In our study, the prevalence of intrusive symptoms, avoidance symptoms, and hyperarousal symptoms among HCWs was 44.2%, 31.8%, 37.7%, respectively, indicating a higher degree of avoidance and hyperarousal symptoms in our study. This difference, more over to the necessity to pay attention to the differences in the tools used in the two studies, could reveal the higher levels of these symptoms among HCWs in our study than in the Yin et al study. Also, the difference may be due to further restrictions on the provision of personal protective equipment for HCWs in Iran, especially in the early stages of the virus's spread due to economic challenges. Also, in both studies, the HCWs who had the most tangency with patients were at higher risk for having PTSS symptoms.

In a multinational and multicenter study, related to HCWs treating COVID-19, 3.8% of patients, experienced moderate to severe levels of psychologic distress, including PTSS. The authors of this paper reported that lethargy and headache were the most common physical or accompanying symptoms in the field of psychologic disorders associated with COVID-19. Among the most important of these disorders were anxiety and PTSD. They stressed the need to pay attention to physical complaints among HCWs in the context of psychological disorders (39). 

Regarding the effects of SARS on the mental health status of HCWs, in a qualitative study, their status was evaluated in the first 4 weeks of disease outbreak. HCWs reported fears of contracting an infectious disease and conveying the disease to family members, friends, and colleagues. Uncertainty and stigmatization were the main themes in the interview. Because of stigma, they avoided going to places identified as HCWs. They experienced fear, anxiety, anger, and frustration (40). Also, in our study, stigma was one of the most important mental health problems of HCWs.

In another study examining stigma, HCWs, among SARS epidemics, all feared infection. The group, which itself had a history of SARS infection, was also concerned about other health problems and discrimination. Fear of SARS in this recent group was correlated with posttraumatic stress symptoms. In this regard, intrusion had the highest prevalence and intensity compared to other domains (41). In our study, intrusion also had the highest prevalence among different domains.

A study examining the state of stigma and its domains during the SARS epidemic and using a tool similar to the one we used in the study, reported a clear difference between intrusion, avoidance, and hyperarousal subscales between individuals in frontline and non-frontline position (10). In our study, too, there was a notable discrepancy between the 2 groups in terms of stigma status and front-line individuals were in a worse condition.

In our study, stigma was more prevalent among physicians and medical residents than in other occupational and employment groups, which could be due to different working conditions. Many physicians and medical residents are in the first line of work due to their job status and are also exposed to more stigma. In addition, physicians seem to be most responsible for caring for patients and are more likely to be addressed than other occupational groups.

In some studies, the link between PTSD and stigma has been discussed, and stigma has been mentioned in addition to some mental health disorders, such as schizophrenia, as a feature associated with PTSD. In our study, there was a positive and strong linear correlation between stigma and PTSD. The same is true for PTSD dimensions, including intrusion, avoidance, and hypervigilance. 

In a study in this regard, Bonfils et al reported high levels of stigma among patients with PTSD. Also, they emphasized the risk of PTSD and comorbidities such as depression due to stigma (42). Stigmatization by the individual's family and society, in the context of trauma itself can act as a factor in the continuity of the PTSS. This effect has been reported beyond the severity of trauma. In fact, stigmatization aggravates PTSD psychopathology and prevents symptoms from improving (43). 

The experience of PTSD symptoms, in addition to the external aspects, which are in the form of negative attitudes of society and others to the individual, can manifest itself along with internal stigma and in the form of negative beliefs about oneself as a result of PTSD symptoms. This can be an important barrier to finding a cure for the symptoms of the disorder and can lead to exacerbation of PTSD (44). 

## Limitation

We navigated this study early in the outbreak of COVID-19 in Iran. The HCWs at that time were under a lot of pressure and were experiencing completely new working conditions. Due to the lack of such studies in the past, implementers and participants had little information about this method of collecting information. On the other hand, the study was conducted at a large number of hospitals. Relevant coordination in this regard, especially due to the limitations related to the conditions of relative quarantine and New Year coincidence in Iran, was accompanied by many difficulties in the implementation of the study.

## Conclusion

The prevalence of PTSS is high among HCWs during COVID-19 pandemic and some demographic characteristics, including gender, working in frontline and hiring status of HCWs, was associated with prevalence of PTSD subscales. Moreover, a strong and significant correlation was observed between stigma score with PTSD total score and its subscales, including intrusion, avoidance, and hypervigilance among HCWs. 

Stigma is also more common among females, front line HCWs, physicians, and medical residents. HCWs are exposed to PTSS and stigma during COVID-19 pandemics. This situation should be considered by health policymakers so that while trying to control the disease, the mental health status of this group of personnel should be given serious attention.
